# Workload Indicators of Staffing Need (WISN) Method for Midwives Planning and Estimation at Asrade Zewude Memorial Primary Hospital, North west Ethiopia

**DOI:** 10.1007/s44250-022-00013-7

**Published:** 2023-01-10

**Authors:** Gizew Dessie Asres

**Affiliations:** 1 Hospital Performance Monitoring and Evaluation Team Leader, Asrade Zewude Memorial Primary Hospital, Bure, Amhara Region Ethiopia; 2 Licensed Consultant on Health and Health Helated Issues, Gizew Pharmacy, Bure, Amhara Region Ethiopia

**Keywords:** Ethiopia, Health workforce, Midwives, Workload indicators of staffing needs (WISN)

## Abstract

**Background:**

Workforce is a crucial component of the health service delivery system. Ethiopia faces health workforce challenges when it comes to evidence based health workforce planning.

Workforce planning was initially determined by comparing the health worker ratio to the general population number. Later, it was determined by standard staffing schedules for each health facility level. However, neither of these methods addressed the evidence based workload variation issue among the same level facilities all around the country. A workload indicator of staff needs (WISN) method can address these variations. Therefore this research was carried on to determine workload pressure excess or gap in midwives, thereby to promote the WISN use in health facilities, based on WISN results of midwives at Asrade Zewude memorial Hospital.

**Methods:**

A cross sectional study using WISN model was used to determine the workload excess and gap pressure in midwives at Asrade Zewude Memorial primary hospital, North West Ethiopia. Midwives were selected based on a priority point scale as outlined in the WISN method.

**Results:**

According to the data obtained, midwives worked five days a week and 1030 h per year. This working time was spent on health service activities (58.4%), additional activities (36.6%) and support activities (5%). WISN calculations demonstrated a shortage of five midwives with WISN ratio of 0.8 at Asrade Zewude Memorial primary hospital North West Ethiopia.

**Conclusion:**

Midwives at the study area were carrying on their routine tasks even though there was a staff gap of 5: thus, the midwives had a workload excess of 20%. Under these conditions, it may be hard for the facility to achieve universal health service goals. Therefore the hospital should institutionalize WISN method planning to objectively employ midwifery professionals. This study had limitations too as it used retrospective annual service statistics and small sample size which affects generalization of the results to other health facilities and other health worker cadres within the study hospital.

## Introduction

All countries, regardless of their socioeconomic level, struggle about healthcare workers' education, employment, performance and retention. Health priorities of sustainable development goals cannot be achieved if not supported by health care work planning and deployment strategies [1, 2].

Countries have been estimating health care workers of a health facility for a long time. The need for tools that facilitate this estimation, including objective and scientific methodologies has grown over time [3–5]. In order to improve health care delivery, health service managers are faced with increasing challenges in finding qualified health care workers that can meet the community expectations. As one of the six building blocks of the WHO framework for health systems, human resources for health (HRH) is one of the most critical factors for a minimum health service package delivery [6].

Organizations need to identify the most appropriate staffing levels and skill mix to ensure the most efficient use of the already limited resources. Often, this is not the case as it is not easy to find qualified health workers in specific geographical areas or health facilities where they are needed most; or in surplus where need is low resulting in inefficiencies. Poor planning and management of human resources for health contribute to these deficiencies. The required number of health workers and their professional diversity in a health facility depends on the workload and the minimum health care package of the facility [3, 7].

Application of Workload Indicators of Staffing Need (WISN) method for health care worker planning and projection helps to rectify many of the observed deficiencies in access to human resource for health. This implies that a health facility has its own staffing requirement which depends on its client load. Following its development by WHO, the WISN has been used to determine staffing requirements in different countries such as Kenya, Sudan, Hong Kong, Papua New Guinea, Sri Lanka, the United Republic of Tanzania, Bahrain, Egypt, Oman and Turkey among others [3, 4, 6, 8–18]. A previous WISN method study on midwives in different parts of the world showed WISN ratio of 0.71–6.09. Workload pressure of midwives was 0.71–6.09 in the Philippines, 1.00 in Guinea and 0.83–1.83 [11, 19, 20] in Greece.

As a human resource management tool, WISN has significant advantages for stakeholders. Human resource managers use WISN for evidence based estimation of the health workforce. Researchers and policy makers consider recommendations for a policy change, implemented on on WISN results of health facilities [21]. The health facilities use WISN to understand the level of workload pressure, the cause of shortage or surplus and staff reassignment [20, 22]. During the monitoring and the evaluation of the health system, WISN can be used for various purposes like enhancing human resources for health investment [23, 24]; updating the system based on current sanctioning norms; managing task division among employees; managing health workforce; observing details in the work process that were not perceived as a source of work overload [25–27]. Most importantly, WISN can also be used for data quality improvement, health service quality improvement, staff satisfaction and patient satisfaction by matching each employee’s skills and competences with the right tasks in order to deliver a high-quality health service [28].

Ethiopia is currently facing challenges in re-confirming health care workers’ contracts for problems related to availability, quality, accessibility, acceptability and effective coverage mainly due to poor health care planning. The population ratio to health worker method and standard staffing schedule for each health facility level are no longer effective for evidence based health care worker planning. Therefore the WISN method of staff requirement to health facilities should be advocated as an option by applying it at Asrade Zewude Memorial primary hospital.

## Methods

A cross sectional WISN design was used to compare the number of available midwives to the number of required midwives across the facility. Midwives have been selected based on priority score using WISN method as explained in detail in Table 1. The gap/excess and workload pressure of midwives were calculated at Asrade Zewude Memorial primary hospital, North West Ethiopia. The hospital is located in the Amhara regional state and located 410 km from Addis Ababa—the capital city of Ethiopia, 155 km from Bahir Dar—the capital city of Amhara region and 30 km from Finoteselam—the capital city of West Gojjam Zone. It has officially been opened on the 19th of September, 2015 and since then it has been serving for Sekela woreda, Womberma woreda, Bure Zuria Woreda and Bure City administration communities with the health work force of 210 employees.

**Table 1:** Determining priority health worker categories for WISN study at Asrade Zewude Primary hospital, 2020 (Customized from WHO WISN user’s manual 2010 [29])

Service unit	Health worker category	Staffing problems (current and likely in the future)	Priority point scale 0 to 10
Emergency	Doctors	We have adequate number of day time Doctors	0
	Integrated Emergency Surgical Officer(IESO)	Small, but not serious, shortage of IESOs in the hospital in the Emergency Room during working hours	5
	Nurses	Small, but not serious, shortage of nurses in the hospital in the Emergency during working hours	6
	Anesthetist	Small, but not serious, shortage of Anesthetists in the hospital in the Emergency during working hours	4
IPD	Doctors	We have adequate number of day time doctors	2
	Integrated Emergency Surgical Officer	Small, but not serious, shortage of IESOs in the hospital in the inpatient ward during working hours	5
	Nurses	Small and serious, shortage of nurses in the hospital in the inpatient wards	7
	Mid wives	Small and serious, shortage of midwives in the hospital in the inpatient wards	9
	Laboratory	Small, but not serious, shortage of laboratory professionals in the hospital in the inpatient ward during working hours	7
	Pharmacy	Small, but not serious, shortage of pharmacy professionals in the hospital in the inpatient ward during working hours	7
	Anesthetist	Small, but not serious, shortage of Anesthetists in the hospital in the inpatient ward during working hours	4
OR	Integrated Emergency Surgical Officer	Small, but not serious, shortage of IESOs in the hospital in the OR during working hours	5
	Doctors	We have adequate number of day time doctors	3
	Nurses	Small, but not serious, shortage of nurses in the hospital in the OR during working hours	5
	Midwives	Small and serious, shortage of midwives in the hospital in the OR	9
	Anesthetist	Small, but not serious, shortage of anesthetists in the hospital in the inpatient ward during working hours	4
OPD	Doctors	We have adequate number of doctors	5
	Integrated Emergency Surgical Officer	Small, but not serious, shortage of IESOs in the hospital in the OPD	5
	Nurses	Small, but not serious, shortage of nurses in the hospital in the OPD during	7
	Mid wives	Small and serious, shortage of midwives in the hospital in the OPD	10
	Laboratory	Small, but not serious, shortage of laboratory professionals in the hospital in the OPD	7
	Pharmacy	Small, but not serious, shortage of pharmacy professionals in the hospital in the OPD	7
	Radiographers	Small, but not serious, shortage of radiographer in the hospital in the OPD	4
NICU	Doctors	We have adequate number of day time doctors	
	Integrated Emergency Surgical Officer	Small, but not serious, shortage of IESOs in the hospital in the NICU	5
	Nurses	Small but not serious, shortage of nurses in the hospital	8
	Mid wives	Small but not serious, shortage of midwives in the hospital	5
Labor and delivery	Midwives	Small but not serious, shortage of midwives in the hospital	9
	Integrated Emergency Surgical Officer	Small, but not serious, shortage of IESOs in the hospital	5
	Doctors	We have adequate number of doctors	1
Highest Priority score cadres: 1. Midwives first highest priority. 2. Nurses second highest priority. 3. Laboratory and pharmacy third highest priority			

The selection of midwives was a priority due to current staffing problems. Their working time availability were determined using the national calendar, the personnel files and the HR regulatory documents. Workload components of midwives were determined by establishing an expert working group, by monitoring their performance and evaluating the unit and the human resources management staff. The expert group took a two hours long detailed training course, using the WISN manual.

The data used by expert working group: detailed job descriptions obtained from human resources management, annual service statistics obtained from hospital performance monitoring and evaluation unit and midwifery service activities and support activities. The workload data were cross checked from July 1, 2019–June 30, 2020 for the midwifery department using health service activity data of the health information system district’s (DHS2) database.

The WISN tool uses information to determine staffing requirements in health care settings. The information includes routine activities performed by midwives at Asrade Zewude Memorial Primary Hospital (i.e., workload components), the necessary time to conduct core activities and associated activities (i.e., activity standards), working time availability in 1 year for the execution of their task and the annual service delivery statistics in the selected health service delivery point [5, 21].

### Determining priority cadres

The WISN tool can be used to determine the number of health care workers of all categories at any health care setting although it is not a budget-friendly option to do it all at one time. The author decided to prioritize which staff categories at Asrade Zewude Memorial Primary Hospital would be the first WISN target (see Table 1). The author preferred to start with the cadres with less complex activity standards. It is then easy to expand this to other health workers in subsequent WISN applications, after the team gained experience with the method.

The author set out priorities in such a way that all the working units have been listed with the staff categories working there. Then, the author detected the most difficult staffing problems regarding these staff cadres. By considering current and future staffing problems, the author decided which staff category (or categories) should have highest priority. Here are some questions to consider in the cadre selection process:

- Which cadre faces supply problems
- Which problem has highly influenced health care quality?
- Which problem is more likely to have an immediate effect on health care quality?
- Are any of the cadres particularly important for future health programs?

## Results

Based on this study, the current employees assigned by the standard staffing schedule (stable number of health workers for the same level of health facility, locally called form 15) and the required staff based on current service standard showed a discrepancy. Number of midwives during data collection was 20 but the required number of midwives based on WISN calculation was 25 so only 80% of the staff needed was available.

The working days per week were five and the working hours per day was 7.8. The annual and other leave-days were 25 and total annual working time was 1030 h (see Table 2).

**Table 2:** Available Working Time (AWT) for Midwives at Asrade Zewude Memorial primary hospital, 2020 G.C

Working days per week (A)	Working hours per day (B)	Annual and other leaves days (C)	Public holidays (D)	Training days per year (E)	AWT in weeks per year (F = A*B)	Total working days per year(G)	Awt in days per year (H = G − (C + D + E))	Awt in hours per year (I = H*B)
5	7.8 (39/5)	25 days	6	82	49	245	132	1030

Source: Asrade Zewude Memorial Hospital Human resource department

Activity standards for midwives working at Asrade Zewude Memorial Hospital were fifteen health service activities, four support activities and five additional activities (see Table 3).

**Table 3:** Activity standards for midwives working at Asrade Zewude Memorial Hospital, 2020 G.C

Workload group	Workload component
Health service activities of all midwives	1. Cervical cancer screening
	2. Short term family planning service
	3. Long term family planning service
	4. First Ante natal care
	5. fourth Ante natal care
	6. labour with partograph without episotomy
	7. Labour with phartograph with Episotomy
	8. Preparing laboring mothers to Cesarian section/ OR
	9. Post Natal Care
	10. Recovery
	11. Safe Abortion
	12. Post abortion care
	13. Immunization
	14. High risk mothers care from admission to follow up per day
	15. Birth notification
Support activities of all midwives	1. Documentation to registration and Tally 10 min per client
	2. Meetings 1 h/2 weeks
	3. Health center mentorship 8 h 2 midwives/16 health center/2 month
	4. Accompanying reoffered out laboring mothers 8 h/client
Additional activities of certain midwives	1. Participating Management meeting 3 h/week
	2. Schedule posting or task assignment of midwives 30 h/month
	3. Reporting performances of the unit 8 h/month
	4. Evaluating performances of each mid wife/PA 5 days/6 month
	5. Reporting monthly attendances for HRM unit 10 min/month

Source: Asrade Zewude Memorial Hospital midwifery department

Based on expert working group observation, attending labor with partograph and doing episiotomy took 12 h. Attending labor with partograph without episiotomy took 8 h. Health service activities requiring a shorter time were family planning, preparing mothers for cesarean section, immunization at birth, postnatal care and birth notification (see Table 4).

**Table 4:** Service standards and annual service statistics of midwifery related activities at Asrade Zewude Memorial Primary Hospital, 2020 G.C

Health service activities	Unit time or rate of working	Annual service statistics	Standard workload
1. Cervical cancer screening and treatment	60 min	65	1030
2. Short term family planning service	20 min per client	949	3090
3. Long term family planning service	30 min per client	312	2060
4. First Ante natal care	60 min	973	1030
5. fourth Ante natal care	30 min	469	2060
6. labor with partograph without episiotomy	8 h	1481	129
7. Labour with partograph with Episiotomy	12hrsper client	506	86
8. Preparing laboring mothers to Cesarean section/ OR	20 min per client	291	3090
9. Post Natal Care	20 min per client	1953	3090
10. Recovery	6 h	291	172
11. Safe Abortion	2 h	25	515
12. Post abortion care	3 h	221	343
13. Immunization at birth	5 min	3039	12,360
14. High risk mothers care from admission to follow up per day	40 min	456	1545
15. Birth notification	3 min	1951	20,600

Source: Asrade Zewude memorial Hospital DHIS2 data

The support activities account for 5% of the total working hours of which 3% is spent on meetings (see Table 5).

**Table 5:** Support activities of midwifery staff at Asrade Zewude Memorial Primary Hospital, 2020 G.C

Workload Components	CAS (actual working time)	CAS % (percentage working time)
1. Documentation to registration and Tally	10 min/day	2% = (10/60)/7.8*100
2. Meetings	2 h/2 weeks	3% = 2 h/(7.8 h*5 days*2wks))*100
Total CAS %		5%

Source: Asrade Zewude Memorial Hospital Human resource and midwifery department

Midwives at Asrade Zewude Memorial Primary Hospital spend a total of 377 h. in a year for additional activities. Additional activities requiring more time were evaluating or performance appraisal of the unit, health center mentorship and preparing performance reports; reporting monthly attendances and schedule posting required a shorter amount of time (see Table 6).

**Table 6:** Additional activities of certain midwives at Asrade Zewude Memorial Primary Hospital, 2020 G.C

Workload components	Number of staff performing the work	IAS (actual working time per person)	Annual IAS (for all staff performing activity)
1. Participating Management meeting	1	2 h 49 times a year	98 h
2. Schedule posting or task assignment of midwives	1	30 min, 12 times a year	6 h
3. Reporting performances of the unit	1	8 h 12 times a year	96 h
4. Evaluating performances of each mid wife/PA	1	5 days Two times a year	80 h
5. Reporting monthly attendances for HRM unit	1	5 min, 12 times a year	1 h
6. Health center mentorship	2	8 h Six times a year	96 h
Total IAS in a year			377 h

Source: Asrade Zewude Memorial Hospital Human resource and midwifery department

Calculation based on WISN showed that about twenty five midwives were needed for health service activities, support activities and additional activities (see Table 7).

**Table 7:** Determining staff requirements based on WISN for midwives at Asrade Zewude Memorial Primary Hospital, 2020 G.C

	Workload component	Annual workload	Standard workload	Required number of staff members
Health service activities of all cadre members	1. Cervical cancer screening	65	1030	0.63
	2. Short term family planning service	949	3090	0.31
	3. Long term family planning service	312	2060	0.15
	4. First Ante natal care	973	1030	0.94
	5. fourth Ante natal care	469	2060	0.23
	6. labor with pantograph without episiotomy	1481	129	11.48
	7. Labour with partograph with Episiotomy	506	86	5.88
	8. Preparing laboring mothers to Cesarean section/ OR	291	3090	0.09
	9. Post Natal Care	1953	3090	0.63
	10. Recovery	291	172	1.69
	11. Safe Abortion	25	515	0.05
	12. Post abortion care	221	343	0.64
	13. Immunization	3039	12,360	0.25
	14. High risk mothers care from admission to follow up per day	456	1545	0.29
	15. Birth notification	1951	20,600	0.09
A. Total required staff for health service activities				23.35
Support activities of all cadre members	Workload component	CAS (Actual working time)	CAS (Percentage working time)	
	Documentation to registration and Tally	10 min/day	2% = (10/60)/7.8*100	
	Meetings	2 h/2 weeks	3% = 2 h/(7.8 h*5 days*2wks))*100	
Total CAS %			5%	
B. Category allowance factor: {1/[1 − (total CAS percentage/100)]} = 1.05				
Additional activities of certain cadre members	Workload component	Number of staff members performing the work	IAS (Actual working time per person)	Annual IAS (for all staff performing activity)
	Participating Management meeting	1	2 h 49 times a year	98 h
	Schedule posting or task assignment of midwives	1	30 min, 12 times a year	6 h
	Reporting performances of the unit	1	8 h 12 times a year	96 h
	Evaluating performances of each mid wife/PA	1	5 days Two times a year	80 h
	Reporting monthly attendances for HRM unit	1	5 min, 12 times a year	1 h
	Health center mentorship	2	8 h Six times a year	96 h
Total IAS in a year				377 h
C. Individual allowance factor (Annual total IAS / AWT)				0.37
Total required number of staff based on WISN: (A x B + C)				24.89 (⁓25)

Source: Asrade Zewude Memorial Hospital Human resource, midwifery department and survey analysis

## Discussion

This study demonstrated the implementation process of the WISN methodology using midwifery staff data at Asrade Zewude Memorial Hospital. According to this study, the midwives at Asrade Zewude Memorial Hospital had fifteen workload components under health service activity, six workload components under additional activity and two workload components as support activity.

The results demonstrated that there were five working days with 1030 h actual working time per year for midwives at Asrade Zewude Memorial Hospital. This working time was spent on health service activities (58.4%), additional activities (36.6%) and support activities (5%).

According to the study, the traditional way of staffing method, actual number of midwives and WISN method has revealed varied results. Based on Form 15 (fixed number of health worker for the same level of health facility) the number of midwives expected was 8 and the actual number of midwives was 20 but when calculated based on available data, 25 midwives were needed based on the WISN method.

The workload pressure among midwives in the study facility was found to be a WISN ratio of 0.8. This was higher than the results obtained by a research including Ugandan midwives (0.53.-0.67), Bangladeshi nurses (0.69), Indonesia (0.64) and Indian midwives (0.57) but it is lower than those obtained by a study that included Greek midwives (1.33–1.83), Guinean midwives (1.00) and Filipino midwives (0.71–6.09) [9–11, 19, 30, 31]. The variation may be a result of sociocultural and political differences as well as facility service variations but in the case of Bangladesh results, cadre differences should be taken into consideration before interpreting the results.

## Limitations

As a WISN method study, the accuracy of the result was based on the accuracy of the hospital annual service statistics (using DHIS2). However, the annual service statistics were affected by Covid-19 restrictions. Another limitation was the sample size, which focused only on midwives, making its generalizability questionable for other health departments and cadres within the same facility.

## Conclusion

WISN method is useful for evidence based staff requirement estimations. It enables health facilities to work with qualified health workers based on an objective workload evidence. WISN workforce planning method had challenges related to proper documentation and data quality. Therefore institutionalizing this tool enables health facilities to focus and strengthen data quality yet another dimension of the health system.

Based on the WISN output of Asrade Zewude memorial Hospital the following recommendation was forwarded to stakeholders.

- Researchers should consider further WISN study scales at a national level.
- Decision makers at regional health bureau and federal ministry of health should better institutionalize WISN method to have the right number of midwives at the right time.
- Human resource department should advocate the use of WISN in the health facility.
- The planning and health information department should enhance WISN based planning as it encourages performance and data quality.

## Data Availability

Necessary materials may be available upon request.
